# Sub-Threshold Fabrication of Laser-Induced Periodic Surface Structures on Diamond-like Nanocomposite Films with IR Femtosecond Pulses

**DOI:** 10.3390/ma15134506

**Published:** 2022-06-26

**Authors:** Sergei M. Pimenov, Evgeny V. Zavedeev, Beat Jaeggi, Josef Zuercher, Beat Neuenschwander

**Affiliations:** 1 Prokhorov General Physics Institute of the Russian Academy of Sciences, 119991 Moscow, Russia; zavedeev@nsc.gpi.ru; 2 Institute for Applied Laser, Photonics and Surface Technologies ALPS, Bern University of Applied Sciences, CH-3400 Burgdorf, Switzerland; bjaeggi@lasea.com (B.J.); josef.zuercher@bfh.ch (J.Z.)

**Keywords:** diamond-like nanocomposite (DLN) films, femtosecond laser, ablation, spallation, laser-induced periodic surface structures (LIPSS), surface plasmon polaritons

## Abstract

In the paper, we study the formation of laser-induced periodic surface structures (LIPSS) on diamond-like nanocomposite (DLN) a-C:H:Si:O films during nanoscale ablation processing at low fluences—below the single-pulse graphitization and spallation thresholds—using an IR fs-laser (wavelength 1030 nm, pulse duration 320 fs, pulse repetition rate 100 kHz, scanning beam velocity 0.04–0.08 m/s). The studies are focused on microscopic analysis of the nanostructured DLN film surface at different stages of LIPSS formation and numerical modeling of surface plasmon polaritons in a thin graphitized surface layer. Important findings are concerned with (i) sub-threshold fabrication of high spatial frequency LIPSS (HSFL) and low spatial frequency LIPSS (LSFL) under negligible surface graphitization of hard DLN films, (ii) transition from the HSFL (periods of 140 ± 30 and 230 ± 40 nm) to LSFL (period of 830–900 nm) within a narrow fluence range of 0.21–0.32 J/cm^2^, (iii) visualization of equi-field lines by ablated nanoparticles at an initial stage of the LIPSS formation, providing proof of larger electric fields in the valleys and weaker fields at the ridges of a growing surface grating, (iv) influence of the thickness of a laser-excited glassy carbon (GC) layer on the period of surface plasmon polaritons excited in a three-layer system “air/GC layer/DLN film”.

## 1. Introduction

Femtosecond laser-induced periodic surface structures (LIPSS), brightly called a scientific evergreen [1], remain to be of undiminishing interest with regard to both the LIPSS formation mechanisms and various applications of laser-structured surfaces [2,3,4,5,6,7,8,9,10,11,12]. For diamond-like carbon (DLC) films, femtosecond (fs) laser ablation and LIPSS formation are preceded by a structural transformation (graphitization) in a thin surface layer [13,14,15,16,17]. The surface graphitization changes surface properties and affects the ablation process and periodic surface nanostructuring during multipulse irradiation [3,10,15,16,17]. Generally, the fs-LIPSS formation on DLC films is attributed to the excitation of surface plasmon polaritons (SPPs) in the graphitized layer, responsible for the periodic enhancement of local laser fields and spatial modulation of ablation rates on the film surface [4,7]. The structure of the graphitized DLC surface, where LIPSS are produced, is that of glassy carbon (GC), according to Raman spectroscopy studies [3,10,15]. The surface relief analysis of the laser-irradiated swelled regions gives an estimation of a few hundred nanometers for the graphitized layer thickness [10,13,16]. The presence of such a ‘glassy carbon’ layer makes a dielectric DLC film a two-layer GC/DLC coating with a top conductive layer having identical optical properties and controlling the interference between surface plasmons and incident light. This can qualitatively explain similar ‘LIPSS period-on-fluence’ dependences observed for hydrogenated DLC (a-C:H) films, hydrogen-free tetrahedral amorphous carbon (ta-C) films, and GC plates [10]. For interpretation of experimentally observed LIPSS periods, the calculations of SPP periods at the air/GC and GC/DLC interfaces were reported [4,18], in which an influence of the thickness of a laser-excited GC layer on the properties of SPPs in such a three-layer system (air/GC layer/DLC) was not considered.

Diamond-like nanocomposite (DLN) a-C:H:Si:O films are a class of SiO_x_-containing hydrogenated DLC coatings [19] with unique structural and mechanical properties (high hardness, low residual stress, low friction, etc.) [20,21,22]. Due to low internal stresses [22], DLN films of ~10 μm thick can be deposited on different substrates, making them very suitable for fs-laser surface structuring and fabrication of micro/nanostructured surfaces with novel properties [17,23,24,25,26,27]. Femtosecond-laser interaction with DLN films is characterized by three successive processes—graphitization, spallation, and evaporation, occurring with increasing fluence [16,17], similar to the laser-induced processes in hydrogenated DLC films [28,29]. The role of these processes in nanostructuring, as well as the conditions of LIPSS formation in DLN films, are not studied yet, except for a recent demonstration of the LIPSS on laser-graphitized swelled regions produced with an IR fs-laser [16]. Of interest is the interrelation of the surface graphitization, microspallation effects, and LIPSS formation during fs-laser processing of DLN films at low fluences, i.e., below the single-pulse ablation threshold, when LIPSS are usually formed.

In this paper, we study the formation of LIPSS on DLN films during nanoscale ablation processing at low fluences—below the single-pulse graphitization and spallation thresholds—using an IR fs-laser (wavelength 1030 nm, pulse duration 320 fs, pulse repetition rate 100 kHz, scanning beam velocity 0.04–0.08 m/s). The aim is to establish the relationships between the irradiation conditions, surface graphitization, LIPSS formation, and spatial characteristics of the periodic surface nanostructures. The studies are focused on microscopic analysis of the nanostructured DLN film surface at different stages of the LIPSS formation, estimation of the surface plasmon fields at early stages of the DLN surface nanostructuring, and numerical modeling of surface plasmon polaritons in a thin graphitized surface layer. New results of sub-threshold fabrication of fs-LIPSS on the DLN film surface, visualization of equi-field lines by ablated nanoparticles at an initial stage of the LIPSS formation, and influence of the thickness of a laser-excited GC layer on the period of SPPs in a three-layer system (air/GC layer/DLN) are presented and discussed.

## 2. Materials and Methods

The DLN films on Si substrates, produced by plasma-assisted chemical vapor deposition from polymethylphenylsiloxane vapors, were used in fs-laser processing experiments. The DLN films of 2.5-μm thickness were characterized by the hardness 27 GPa, elastic modulus 143 GPa, and compressive stress 268 MPa. All details about the deposition technique and mechanical properties of the films are given in our recent paper [22]. The optical properties of the DLN films, including the refractive index *n* = 2, extinction coefficient k_a_ = 0.021, and absorption coefficient α = 2.6 × 10^3^ cm^−1^ at the laser wavelength λ = 1030 nm, were reported in our previous article [17].

Laser ablation processing of the DLN films was carried out using a SATSUMA HP2 femtosecond laser system [30,31] (from Amplitude Systèmes, Pessac, France), generating pulses of τ = 320 fs duration at the wavelength λ = 1030 nm. In the experiments, the average power (*P*) was varied from 100 to 154 mW at the pulse repetition rate (*f*) 100 kHz; the pulse energy (*ε_p_*) was changed from 1 to 1.54 μJ. The laser beam was focused with a 160-mm f-theta objective, and the beam radius (*w_o_*) of 17.5 µm at the 1/e^2^ level was determined with a scanning slit beam profiler from Thorlabs (Newton, NJ, USA). For the used pulse energies, the peak fluence *F =* 2*ε_p_*/*πw_o_*^2^ was ranged from *F* = 0.21 to 0.32 J/cm^2^, with these values being smaller than the single-pulse graphitization and spallation thresholds—*F_gr_* = 0.3 J/cm^2^ and *F_spal_* = 0.35 J/cm^2^ [17]. A high-precision galvanometer scanner intelli*SCAN*_se_ from Scanlab (Puchheim, Germany) was applied to control the scanning beam velocities (*v_s_*) and to produce linear surface microstructures (microgrooves) of 10–20 μm width and 15 mm length at different irradiation conditions. The microgrooves were produced at the fluences increasing from *F* = 0.21 to 0.32 J/cm^2^, two different scanning velocities of 0.08 m/s and 0.04 m/s, and the pulse repetition rate of 100 kHz. For the two scanning velocities, the pitch distance (*v_s_*/*f*) was 0.8 µm and 0.4 µm, respectively. The ablation rate was determined as the groove depth divided by the effective pulse number *N = w_gr_*/(*v_s_/f*) (*w_gr_* is the groove width). The laser beam was linearly polarized and the direction of the beam polarization was perpendicular to the scanning direction. All laser processing experiments were carried out in ambient air at the normal beam incidence onto the film surface.

The dimensions and surface morphology of the microgrooves were examined using scanning electron microscopy (SEM), white-light interferometry (WLI), and atomic force microscopy (AFM). The data of the DLN structure transformation in a thin surface layer was obtained from Raman spectra measured at the excitation wavelength 532 nm. The two-dimensional Fast Fourier Transform (FFT) analysis of SEM and AFM images was applied to determine the periods of LIPSS.

## 3. Results and Discussion

**Figure 1:**
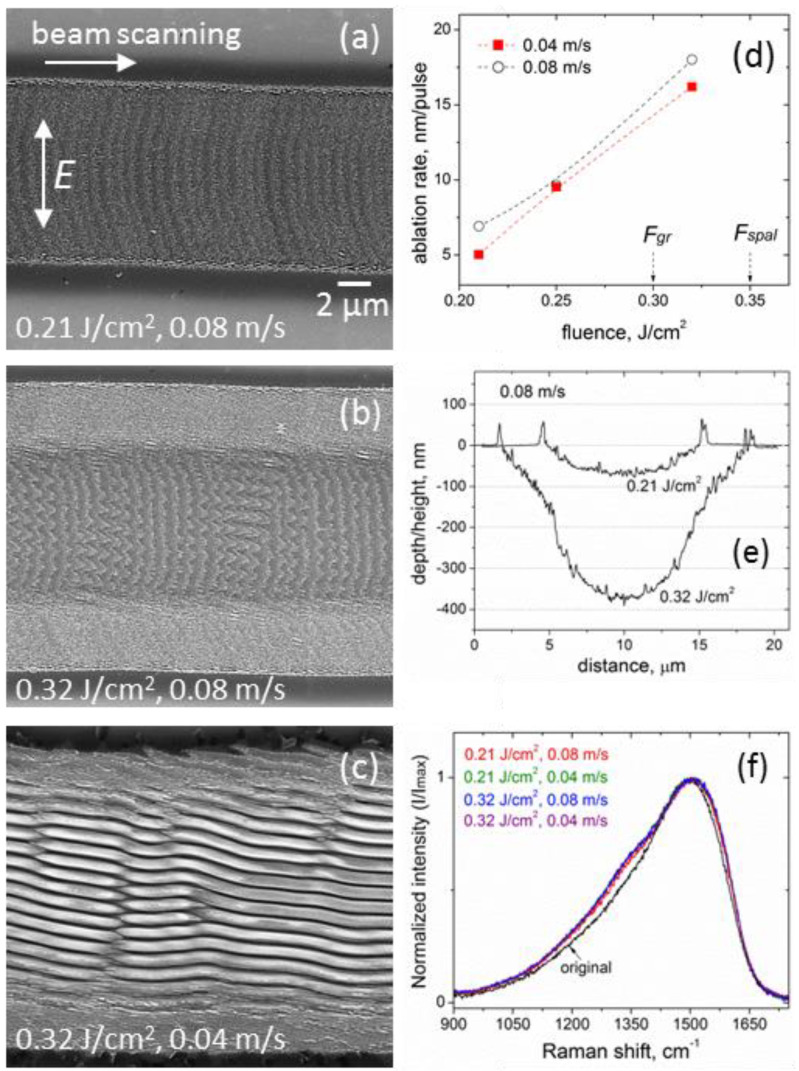
SEM images of microgrooves produced on the DLN film with a scanning beam of the IR fs-laser at (**a**) *F* = 0.21 J/cm^2^ and V_s_ = 0.08 m/s, (**b**) *F* = 0.32 J/cm^2^ and V_s_ = 0.08 m/s, (**c**) *F* = 0.32 J/cm^2^ and V_s_ = 0.04 m/s, the polarization direction is perpendicular to the beam scanning direction; (**d**) the ablation rate vs. fluence for the two scanning velocities, arrows indicate the single-pulse graphitization and spallation thresholds; (**e**) AFM surface profiles of the microgrooves shown in (**a**,**b**); (**f**) Raman spectra (normalized to the maximum intensity) from central regions of the microgrooves, produced at *F* = 0.21 J/cm^2^ and *F* = 0.32 J/cm^2^ and two scanning velocities of 0.08 and 0.04 m/s.

SEM images of microgrooves produced at low fluences (below the *F_gr_* and *F_spal_* thresholds) and two scanning velocities are shown in Figure 1, along with the ablation characteristics (Figure 1d,e) and Raman spectra (Figure 1f) of the grooves.

**Figure 2:**
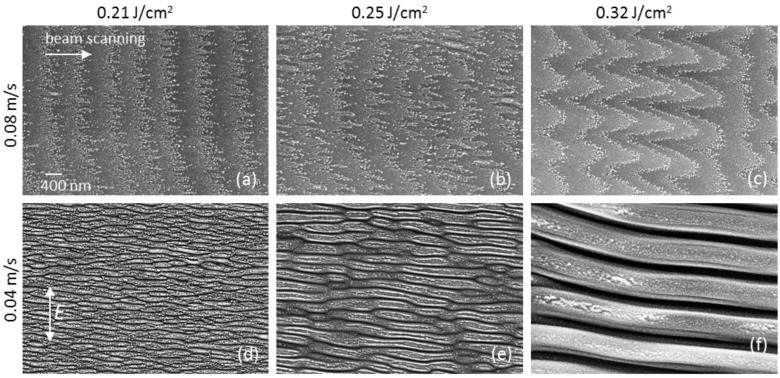
Changes in the surface morphology in central regions of the microgrooves on the DLN film with increasing fluence (0.21 J/cm^2^, 0.25 J/cm^2^, and 0.32 J/cm^2^) at two scanning velocities of 0.08 m/s (**a**–**c**) and 0.04 m/s (**d**–**f**); the beam polarization direction is perpendicular to the scanning direction.

Changes in the groove morphology with increased fluence at the *v_s_* = 0.08 m/s, shown in Figure 1a,b, evidence an appearance of the relief periodicity, which is transformed to LIPSS (Figure 1c) at the lower velocity *v_s_* = 0.04 m/s and correspondingly larger pulse number. Details of the surface morphology in the central regions of the microgrooves fabricated at different fluences (0.21, 0.25, and 0.32 J/cm^2^) and two scanning velocities are presented in SEM images of higher magnification in Figure 2. These images confirm the formation of LIPSS at all the fluences and low scanning velocity *v_s_* = 0.04 m/s, attributing the surface relief changes at the higher velocity *v_s_* = 0.08 m/s (lower effective pulse number) to an initial stage of LIPSS formation.

**Figure 3:**
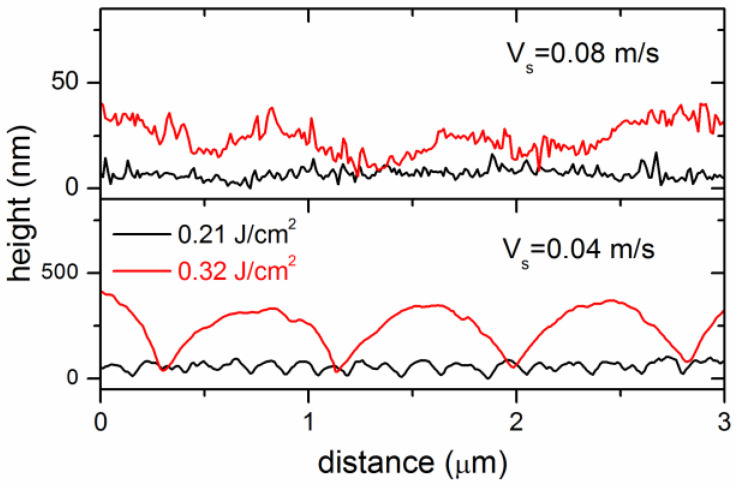
AFM surface profiles were measured in the central regions of microgrooves produced at two fluences (0.21 J/cm^2^ and 0.32 J/cm^2^) and two scanning velocities (AFM tip scanning was made across the grooves perpendicularly to the beam scanning direction).

Additionally, AFM examination of the grooved surface provides information on the depth of the formed surface gratings. The AFM surface profiles, measured in the central regions of the microgrooves produced at the minimal and maximal fluences, are shown in Figure 3. For *F* = 0.21 J/cm^2^, the depth of the nanograting formed at *v_s_* = 0.04 m/s is about 50 nm, while it is of the order of several nanometers at the early stage of the LIPSS growth (not clearly pronounced in the profile). For *F* = 0.32 J/cm^2^, shallow ripples of 10–15 nm depth are formed at the *v_s_* = 0.08 m/s compared to the 300 nm depth of the surface grating fabricated at the low velocity *v_s_* = 0.04 m/s.

The results of SEM, AFM, and Raman spectroscopy examination of the microgrooves show a number of interesting features of sub-threshold ablation processing with an IR femtosecond-laser. At all fluences used, the fs-laser processing and periodic surface nanostructuring of the DLN film occur without noticeable surface swelling arising from the surface graphitization [10,16]; sharp swellings of ~50 nm height seen in the surface profiles in Figure 1e are due to protruding thin layers caused by their spallation and fracture at the groove edges. It is different from the previous data of fs-LIPSS in DLN [16,17] and ta-C [10] films with the LIPSS formed on swelled graphitized regions of the film surface. These findings are consistent with the Raman spectra shown in Figure 1f, which indicate minor changes in the structure of laser-graphitized surface layers with increased fluence/pulse number and very weak surface graphitization during sub-threshold processing and LIPSS formation. The spectrum of the original film is characterized by an asymmetric broad G-peak near 1490 cm^−1^. In the Raman spectra of the microgrooves, one can see only an appearance of a ‘shoulder’ of the slightly increased intensity in a region of the D peak around ~1300–1350 cm^−1^ and hardly observable changes near the G-peak. The observed minor changes in the spectra evidence that the thickness of a laser-graphitized surface layer is very small. Using the procedure of the original film spectrum subtraction described in the recent papers [26,32], we estimate the graphitized layer (GL) thickness of <10 nm on the groove surface under the processing conditions studied. The fs-laser processing without noticeable surface graphitization is indicative of a thermo-mechanical mechanism of ablation to be dominant at sub-threshold fluences. This is also supported by the clear evidence of laser-induced spallation on the tops of hill-like regions of the surface gratings, shown in Figure 2d,e, and by the presence of numerous nanoparticles (of a few tens nanometers size) on the surface of microgrooves after processing at the higher scanning velocity, as is seen in the images in Figure 2a–c. The formation of very specific nanostructures from nanoparticles is found to occur at the beginning of the LIPSS growth, which is considered to result from the interference of incident linearly-polarized light with surface plasmons (SP) and variation of local ablation thresholds in valleys and hills of a growing surface grating.

The typical surface morphology inside laser-ablated microgrooves is shown in Figure 1a, also observed in the previous laser structuring experiments of DLN films [23,26]. It is characterized by arc-like curves/regions consisting of nanoparticles and corresponding to the back edges of the laser spot moving in the direction from left to right with the pitch length of *v_s_*/*f* = 0.8 μm. Most interestingly, these arc-like regions are found to transform to sinusoid-like ‘nanoparticle’ curves in the groove central areas at the higher fluence *F* = 0.32 J/cm^2^, shown in Figure 1b and Figure 2c. So they also have been the back edges of the laser spot moving from left to right with a pitch length of 0.8 μm. Similar sinusoid-like structures from nanoparticles are noticeable at lower fluences as well, in Figure 2b and even in Figure 2a, but they are weakly pronounced due to a smaller period of nascent LIPSS and relatively large size (a few tens of nanometers) of nanoparticles.

We assume that the edges of the moving spot ‘show’ the equi-field (equi-fluence) lines coinciding with the ablation (spallation) threshold of the groove surface or the ablation threshold of nanoparticles redeposited in the groove during processing. Under this assumption, we estimate the SP fields using the data of Figure 1a and Figure 2a,c, as described below.

The radial distribution *E_L_*(*r*) of the electric field of the incident laser beam is given by

*E*_*L*_(*r*) = *E*_*0*_*exp*(−*r*^2^/*w*_*o*_^2^) = *E*_*0*_·*exp*(−(*x* + *y*)^2^/*w*_*o*_^2^) (1)

where *E*_0_ is the electric field in the spot center, *w_o_* is the beam radius at the 1/e^2^ level, *x* and *y* are the coordinates parallel and perpendicular to the beam scanning direction. The beam polarization is directed normally to the scanning direction, i.e., along the y coordinate.

The electric field oscillations of the standing wave of SP (along the *y* axis) are described by

*E*_*SP*_(*y*) = *E*_*SP,MAX*_*cos*(2*πy*/*λ*_*SP*_) (2)

where *λ_SP_* is the wavelength of the SP, *E_SP,MAX_* is the amplitude of the tangential component of the electric field (the amplitude of the SP field is assumed constant as the ‘spot edge’ width Δ*r* is much less than its radius *r_th_*, see below). The positive values of *E_SP_*(*y*) mean in-phase oscillations of the SP and laser fields and the negative values mean anti-phase oscillations of the fields.

**Figure 4:**
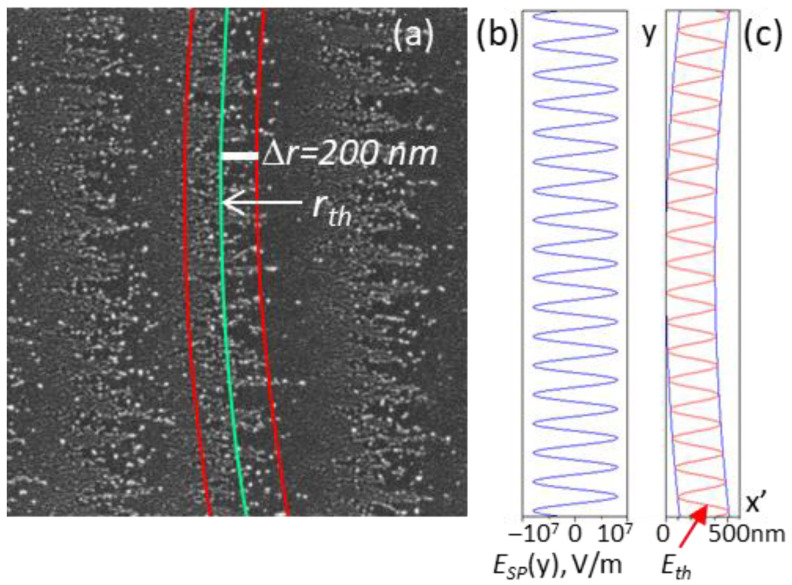
(**a**) A part of the SEM image in Figure 2a, (**b**) electric field oscillations of the SP at *F* = 0.21 J/cm^2^, and (**c**) *E_th_* = *const* is the equi-field sinusoid curve corresponding to the ablation (spallation) threshold inside the groove fabricated at *F* = 0.21 J/cm^2^.

Taking the radius *r_th_* to define the ablation threshold of the groove surface, the threshold laser field in the absence of the SP is determined from Equation (1) as *E_th_* = *E_L_*(*r_th_*) = *E*_0_ *exp*(−*r_th_*^2^/*w_o_*^2^). In the presence of the SP, the radius *r_th_* is the average value between two boundaries at the distances of *r_th_* ± Δ*r*, as marked in Figure 4a by a green curve and two red curves, respectively. This is due to the interference of the laser field with the tangential (parallel to the surface) component of the electric field of the surface plasmon generated in the graphitized layer. Decomposing *E_L_*(*r*) into a Taylor series, we obtain the equation.

*E*_*th*_ (1 − + 2 *r*_*th*_ Δ*r*/*w*_*o*_^2^) ± *E*_*SP*_ = *E*_*th*_ (3)

from which the amplitude of the tangential component of the SP field is determined by

*E*_*SP,MAX*_ = 2 *E*_*th*_
*r*_*th*_ Δ*r*/*w*_*o*_^2^ (4)

The electric field oscillations of the SP are shown schematically in Figure 4b. The interference of the SP and incident linearly-polarized light defines the boundary of the ablation threshold inside the groove by the equation.

*E_SP_*(*y*) + *E_L_*((*x*^2^*+ y*^2^)^1/2^) = *E_th_* (5)

The solution of Equation (5) is *x* = (*w_o_*^2^ *ln*(*E*_0_/(*E_th_ − E_SP_*(*y*)) *− y*^2^)^1/2^; it is shown in Figure 4c by a sinusoid curve in the coordinates *x’ = r_th_ +* Δ*r–x, y*, with the positive *E_SP,MAX_* values corresponding to the ablation threshold field *E_th_* at the (*r_th_ +* Δ*r)* radius and the negative *E_SP,MAX_* values—to the *E_th_* at the (*r_th_ −* Δ*r)* radius.

Taking the radius of the spot edges equal approximately to *r_th_* = 17.5 μm (from Figure 1a) and Δ*r =* 200 nm (from Figure 2a or Figure 4a), we estimate the tangential component of the electric field of the SP generated in the graphitized layer at the lowest fluence *F* = 0.21 J/cm^2^. For the optical properties of the graphitized surface layer taken identical to the GC properties *ε_GC_* = 3.6 + 4.5*i* at 1000 nm [18] and laser pulse duration *τ =* 320 fs, we obtain the following values of (i) laser intensity in the spot center (in the GL) *I*_0_ = (1 − *R*) *F*/*τ* = 5.9 × 10^11^ W/cm^2^, (ii) laser field in the spot center *E*_0_ = (2 *I*_0_/(*c n ε*_0_))^1/2^ = 1.52 × 10^9^ V/m, (iii) *E_th_* = 5.6 × 10^8^ V/m, and (iv) *E_SP,MAX_* = 1.3 × 10^7^ V/m (*R* = 0.1 is the reflection coefficient of the GC surface).

**Figure 5:**
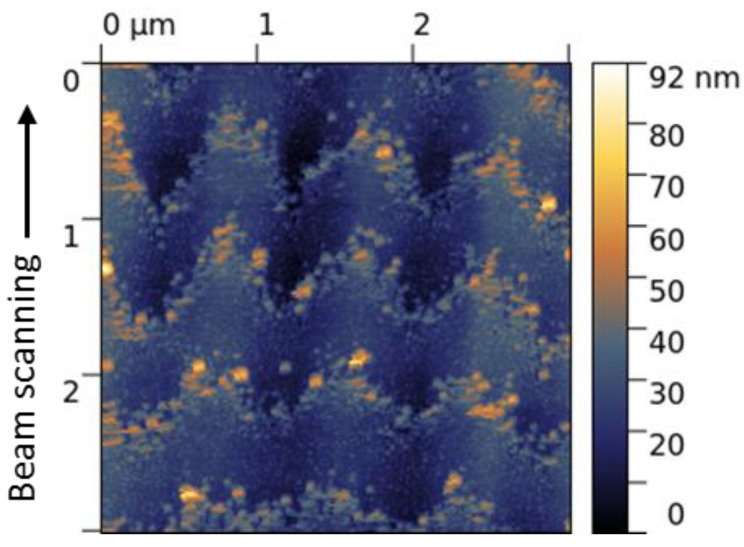
AFM image of a central region in the microgroove produced on the DLN film at *F* = 0.32 J/cm^2^ and *v_s_* = 0.08 m/s and shown in Figure 1b; AFM tip scanning was made across the groove perpendicularly to the laser beam scanning direction.

At the higher fluences, especially at *F* = 0.32 J/cm^2^, the values of Δ*r* increase up to 600 nm, see Figure 2c. According to Equation (4), the SP field is estimated to be more than three times the above value of *E_SP,MAX_ =* 1.3 × 10^7^ V/m, as the *E_th_* remains the same and the *r_th_* increases with fluence. It is seen in Figure 1b and Figure 2c that the amplitude of the equi-field sinusoid-like curves changes along the groove which could be dealt with a variable depth of a growing surface nanograting and enhancement of evanescent electric fields with the grating depth at the corrugated surface [33]. Most importantly, the location of the equi-field ‘nanoparticle’ curves on the emerging surface grating of 10–15 nm depth, shown in an AFM image in Figure 5, proves that the larger local electric fields are in the valleys and the weaker fields are at the ridges of the growing LIPSS. In addition, the observed fluence-dependent transformation of the convex ‘averaged *E_th_*’ curves (related to the back edges of the laser spot, in Figure 1a) to the slightly concave ones (in the central area of the groove, in Figure 1b) is very unusual and not understood. It may be due to the surface relief of the microgroove changing with fluence (Figure 1e) when the effects of the groove slope on the local electric fields should be considered in further analysis and checked in experiments. More particular experimental data on the ablation mechanism and formation of nanoparticles during the sub-threshold processing with IR fs-laser are also required for a better understanding of the LIPSS growth on the DLN surface in dependence on the irradiation conditions.

**Figure 6:**
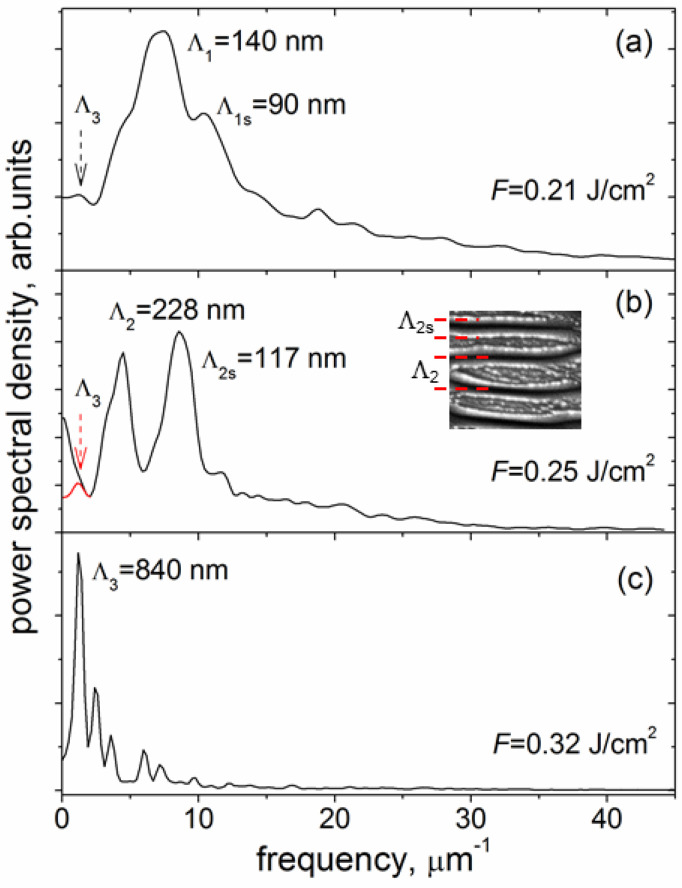
1D-FFT spectra (**a**–**c**) characterizing the periodical nanorelief in the direction perpendicular to the ripples of the SEM images shown in Figure 2d–f; the Λ_3_ peak (marked in red) in (**b**) is a result of the FFT spectrum decomposition in the frequency range of 0–2 μm^−1^; an inset in (**b**) illustrates that the appearance of the Λ_2s_ peak is due to high-brightness edges of thin layers formed by spallation.

The SEM images (in Figure 2) of the LIPSS formed at the *v_s_* = 0.04 m/s evidence that the LIPSS period (Λ) increases with fluence within the range from 0.21 to 0.32 J/cm^2^, very similar to the period-on-fluence dependences for the fs-LIPSS produced on other carbon-based coatings [10]. The data of the LIPSS period was obtained from the FFT analysis of SEM images of 6 μm × 4 μm size and AFM images of 5 μm × 5 μm size. The one-dimensional FFT spectra of the SEM images shown in Figure 2d–f, characterizing the periodical nanorelief on the bottom of the microgrooves, are presented in Figure 6. It is seen that the LIPSS period increases from Λ = 140 nm to Λ = 840 nm, being significantly smaller than the laser wavelength (Λ < λ/7) at *F* = 0.21 J/cm^2^ and becoming close to the laser wavelength at *F* = 0.32 J/cm^2^. Following the proposed terminology of LIPSS [1,12], this means that the change from the high spatial frequency LIPSS (HSFL) to the low spatial frequency LIPSS (LSFL) occurs during sub-threshold processing of DLN films with IR fs-laser pulses in the narrow fluence range under consideration.

**Figure 7:**
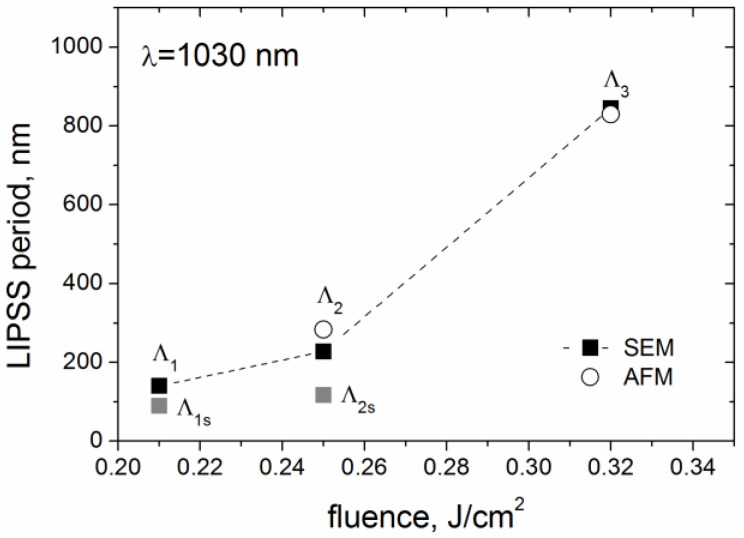
Period vs. fluence of the LIPSS formed on the DLN film during sub-threshold processing with IR fs-laser at the scanning velocity *v_s_* = 0.04 m/s.; designation of the LIPSS periods (Λ_1,_ Λ_2_, etc.) is given in Figure 6.

An interesting feature of the obtained 1D-FFT spectra is related to the appearance of a second peak of higher frequency (smaller period) in the FFT spectra of the SEM images of the groove surface processed at two low fluences, especially in the spectrum shown in Figure 6b. The higher-frequency peak, marked as Λ_2s_ in Figure 6b (and Λ_1s_ in Figure 6a), is due to high-brightness edges of thin layers originating from laser-induced spallation on the tops of hill-like regions of the forming surface grating. This makes the Λ_2s_ (and Λ_1s_) peak not relevant to the true period of the formed LIPSS, as it is defined principally by the yield of secondary electrons but not the surface grating parameters. The data of the FFT analysis of the periodical surface nanostructures formed on the DLN film in sub-threshold fs-laser processing are summarized as the LIPSS period-on-fluence dependence in Figure 7.

**Figure 8:**
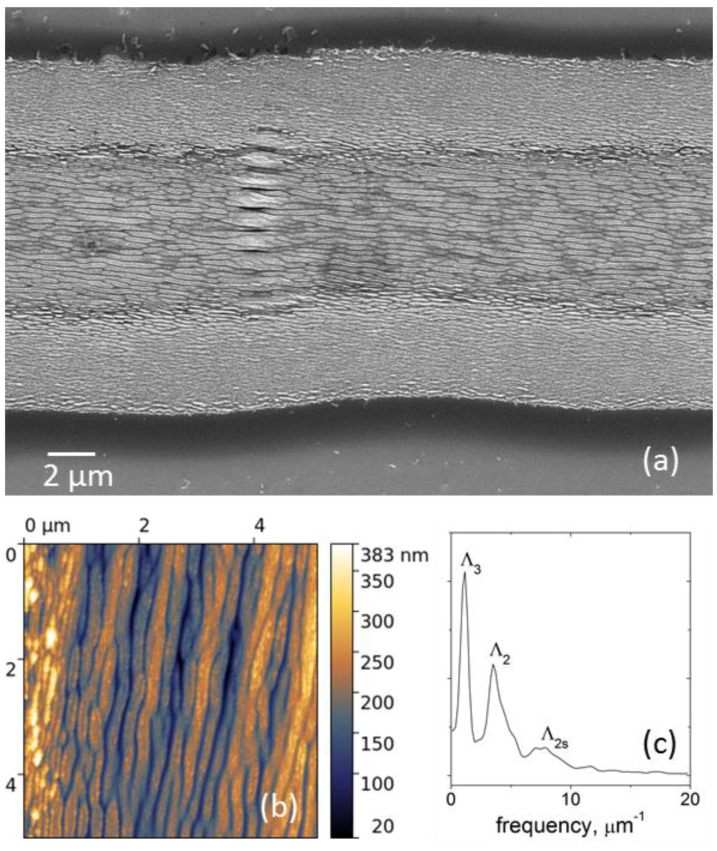
(**a**) SEM image of the microgroove on the DLN film produced at *F* = 0.25 J/cm^2^ and scanning velocity *v_s_* = 0.04 m/s, and (**b**,**c**) AFM image of the groove bottom with a corresponding 1D-FFT spectrum in the direction perpendicular to the ripples.

It should be noted that the low spatial frequency peak (Λ_3_) is present in all the FFT spectra of Figure 6, although weakly pronounced in the spectra in Figure 6a,b for two low fluences. In the frame of the SPP model in multilayer systems [34] (also referred to as a thin-film SPP model [35]), simultaneous observation of the low spatial frequency and high spatial frequency peaks seems to be a consequence of the localization of coupled SPPs at the two interfaces (air/GL and GL/DLN) in a thin graphitized layer on the film surface and fluence-dependent effect of the excited SPP modes on the LIPSS formation. In addition, a superposition of the HSFL and LSFL is found to occur in local regions of the microgroove produced at *F* = 0.25 J/cm^2^ and *v_s_* = 0.04 m/s, as shown in SEM and AFM images and FFT spectrum in Figure 8; the values of the HSFL and LSFL periods are Λ_2_ ≈ 280 nm and Λ_3_ ≈ 900 nm. Whether it is a further proof of the coupled SPPs manifestation or a short-time instability of the pulse energy during beam scanning (e.g., pulse energy increases by 20–30% in several successive laser shots)—both will need more experimental data to clarify the emergence of LSFL under the above lower fluence processing. Generally, the SEM image in Figure 8a is a unique picture of the LIPSS-structured DLN surface. It illustrates the effect of fluence on the HSFL period increasing from the edge to center regions due to the Gaussian intensity distribution and occurrence of the LSFL in local areas of the HSFL-structured surface. The discussed-above ‘artificial’ nature of the Λ_2s_ peak is evidenced as well, as the peak amplitude is strongly reduced in the FFT spectrum of an AFM image which shows the true relief of the surface grating formed on the groove bottom (see Figure 8b,c).

**Figure 9:**
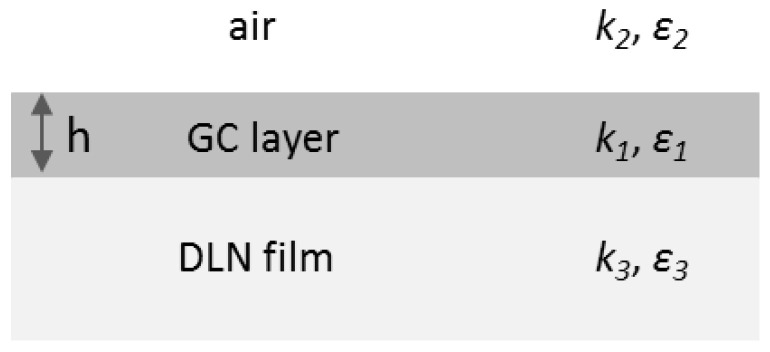
The geometry of a three-layer system used in numerical calculations of the SPP excitation in a thin glassy carbon layer on the DLN film.

For the analysis of fs-LIPSS periods obtained under the low-fluence processing and weak surface graphitization of the DLN film, we applied the SPP model for multilayer systems (consisting of conducting and dielectric layers) [34] to calculate the periods of SPPs excited within a thin graphitized layer of different thickness ranging from 5 to 100 nm. The geometry of a three-layer system, including the air environment (medium 2), graphitized layer of the thickness *h* (medium 1), and DLN film (medium 3), is given in Figure 9. The concentration of the laser-induced plasma (*N_e_*) was assumed to be uniform throughout the entire volume of the graphitized layer, and the DLN electronic subsystem was considered unexcited. The properties of the graphitized layer were taken identical to the optical properties of glassy carbon at λ = 1000 nm [18,36].

The dielectric permittivity of the graphitized layer is given by *ε*_1_(*ω*) *= ε_GC_* − *ω_p_*^2^*/ω*^2^ (for simplicity, the damping of free carriers is ignored), where *ε_GC_* = 3.6 + 4.5*i* is the dielectric constant of the unexcited GC material, *ω* is the frequency of the laser wavelength, *ω_p_* = [*N_e_ e*^2^/(*ε*_0_ *m*)]^1/2^ is the plasma frequency, *e* is the electron charge, *ε*_0_ is the dielectric permittivity of vacuum, *m* = 0.5 *m_e_* is the optical effective mass of electrons. The dielectric constants of air and DLN film are *ε*_2_ = 1 and *ε*_3_ = 4 + 0.084*i*. The excitation of SPPs at the metal-dielectric interfaces is allowed for Re(ε_1_) < 0, Re(ε_2,3_) > 0. The condition of Re(ε_1_) = 0 is fulfilled at *N_e_* =1.9 × 10^21^ cm^−3^ for the laser wavelength λ = 1030 nm, and calculations were carried out for the *N_e_* values from 1.9 × 10^21^ to 1 × 10^23^ cm^−3^.

The SPP wave numbers of a dielectric-metal-dielectric system are described by an expression [34].

(6) $$e^{-4k_{1}a}=\frac{k_{1}/\varepsilon_{1}\;+k_{2}/\varepsilon_{2}}{k_{1}/\varepsilon_{1}-k_{2}/\varepsilon_{2}}\cdot \frac{k_{1}/\varepsilon_{1}+k_{3}/\varepsilon_{3}}{k_{1}/\varepsilon_{1}-k_{3}/\varepsilon_{3}},$$

where $k_{i}^{2}=\beta^{2}-k_{0}^{2}\varepsilon_{i}$ is the component of the wave vector perpendicular to the interfaces in the medium *i* (*i* = 1, 2, 3), *a* = *h*/2 is a half the thickness of a metal layer (i.e., GC layer in the calculations), *β* is the SPP wave number, and *k*_0_ is the wave vector of light in vacuum.

For a → ∞, the Equation (6) transforms to the equation of SPPs at the respective interfaces (air/GC and GC/DLN) between the two half-spaces [34]

(7) $$\beta_{2,3}=k_{0}\sqrt{\frac{\varepsilon_{1}\varepsilon_{2,3}}{\varepsilon_{1}+\varepsilon_{2,3}}}\;.$$

**Figure 10:**
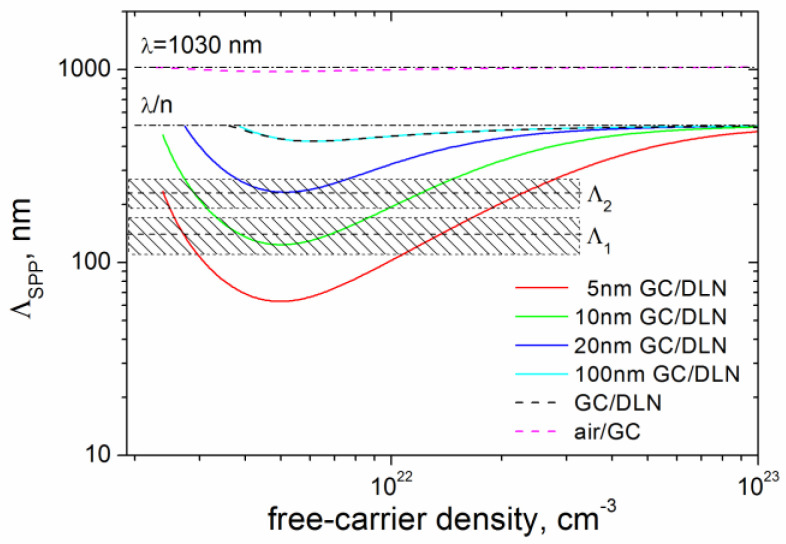
Period of SPP modes vs. free-carrier density, calculated using Equation (6) for different thicknesses of the laser-excited GC layer (5, 10, 20, and 100 nm); for comparison, the Λ_SPP_ data calculated for the interfaces air/GC and GC/DLN using Equation (7) are shown by dashed lines; the Λ_1_ (140 ± 30 nm) and Λ_2_ (230 ± 40 nm) regions mark the experimental periods of LIPSS from Figure 6 and Figure 7; dash-dot lines mark the light wavelengths in air and DLN film (*n* = 2).

Equation (6) was numerically solved for *β* for different thicknesses of the conducting GC layer and optical constants *ε_i_*. Then, the SPP period Λ_SPP_ was calculated as Λ_SPP_ = 2π/Re(*β*) [35]. The results of the numerical calculations for the GC layer thickness of *h* = 5, 10, 20, and 100 nm are shown in Figure 10. The data calculated for the single interfaces (air/GC and GC/DLN) using Equation (7) are also presented for comparison. It follows from Figure 10 that the periods of the HSFL Λ_1_ = 140 ± 30 nm and Λ_2_ = 230 ± 40 nm are in good agreement with the SPP periods for the carrier densities in the range of *N_e_* ~ 5 × 10^21^ − 1.4 × 10^22^ cm^−3^. It is worth noting that the solutions of Equation (6), for which Im(*β*) > Re(*β*), are not considered due to the high absorption of such SPP modes, making their appearance unlikely. For the very thin laser-excited GC layers (of 5–20 nm thick), our calculations show that Im(*β*) > Re(*β*) is observed at *N_e_* < 4.7 × 10^21^ cm^−3^. The LSFL period Λ_3_ = 830–900 nm is found to be close to periods of SPPs localized at the air/GC interface both in the thin-film and single-interface SPP models.

Short remarks about the GL thickness and fs-laser processing at sub-threshold fluences are to be made. The very low GL thickness (<10 nm) together with low ablation rates of 5–16 nm/pulse (which are much smaller than the optical absorption length 1/α ~ 100 nm in the GL at λ = 1030 nm) are indicative of nonlinear absorption of IR fs-laser pulses in the surface layer followed by specific thermo-mechanical ablation process when the heated nm-thick layers are removed from the surface by spallation. (α ≈ 10^5^ cm^−1^ is the absorption coefficient of GC [36]; data on the two-photon absorption coefficient of DLC are lacking in the literature [37]). Indeed, our previous data of numerical simulations of the temperature and surface graphitization in a 2.5-μm-thick DLN film at linear absorption and similar fluence evidenced the heat-diffusion-assisted growth of the graphitized layer under the actions of successive IR fs-laser pulses [17], very different from the results presented in this work. The total thickness of the ablated and graphitized surface layers *h_t_ = h_abl_ + h_gr_* is estimated to be 10–25 nm per laser shot at the minimal and maximal fluences (0.21 and 0.32 J/cm^2^). The *h_t_* is equal to the maximal thermal length, which defines the time of removal (ablation, spallation) of the laser-excited layer as *t_abl_ = h_t_^2^/χ* ~ 0.5–3 ns, consistent with the typical timescales of the material response to fs-laser excitation [38]. So, the ablation rate defines the thickness of the laser-excited graphitized layer which is used in the numerical calculations and responsible for the observed LIPSS periods, while the thickness of the graphitized surface layer (determined after processing) is related to the material properties responsible for characteristics of the ablation process. In our experiments, these values almost coincide, characterizing the sub-threshold ablation processing and LIPSS formation without noticeable surface graphitization.

## 4. Conclusions

The important findings of this work result from distinctive features of sub-threshold processing of DLN films with the IR fs-laser pulses, characterized by nanoscale ablation, spallation, nanoparticle formation, and very weak surface graphitization. The formation/dimensions of LIPSS on the DLN film surface are shown to depend on the laser fluence and scanning beam velocity. Very specific ‘sinusoid-like’ nanostructures from nanoparticles are revealed to form on the ablated surface at an initial stage of the LIPSS formation, considered as a visualization of equi-field lines by nanoparticles due to the interference of incident linearly-polarized light with surface plasmons and variation of local ablation thresholds in valleys and hills of a growing surface grating. The observed location of the equi-field ‘nanoparticle’ curves on the emerging surface grating of 10–15 nm depth can be concluded as a direct experimental proof of the larger electric fields in the valleys and the weaker fields at the ridges of the growing LIPSS. Based on the FFT analysis of SEM/AFM images, it is found that the change from the HSFL (with the periods of Λ_1_ = 140 ± 30 nm and Λ_2_ = 230 ± 40 nm) to the LSFL (Λ_3_ = 830–900 nm) occurs during sub-threshold processing of DLN films in the narrow fluence range of *F* = 0.21–0.32 J/cm^2^. The simultaneous observation of the low spatial frequency and high spatial frequency peaks in FFT spectra indicates the localization of coupled SPPs at the two interfaces (air/GL and GL/DLN) in a thin graphitized layer and the fluence-dependent effect of the excited SPP modes on either HSFL or LSFL formation. The HSFL periods are shown to be in good agreement with numerical calculations of the periods of SPPs excited within a GC layer of 5–20 nm thickness, performed with the SPP model for a three-layer system “air/GC layer/DLN film”. The LSFL period is shown to be close to the periods of SPPs localized at the air/GC interface. The obtained results demonstrate that fabrication of both the HSFL and LSFL with negligible surface graphitization of hard DLN coatings can be realized during IR fs-laser processing at sub-threshold fluences.

## Data Availability

Not applicable.
